# How Do Xanthophylls Protect Lipid Membranes from Oxidative Damage?

**DOI:** 10.1021/acs.jpclett.3c01374

**Published:** 2023-08-14

**Authors:** Renata Welc-Stanowska, Rafal Pietras, Bohun Mielecki, Marcin Sarewicz, Rafal Luchowski, Justyna Widomska, Wojciech Grudzinski, Artur Osyczka, Wieslaw I. Gruszecki

**Affiliations:** † Department of Biophysics, Institute of Physics, Maria Curie-Sklodowska University, 20-031 Lublin, Poland; ‡ Institute of Agrophysics, Polish Academy of Sciences, 20-290 Lublin, Poland; § Department of Molecular Biophysics, Faculty of Biochemistry, Biophysics and Biotechnology, Jagiellonian University, 30-387 Krakow, Poland; ∥ Doctoral School of Exact and Natural Sciences, Jagiellonian University, Prof. Stanisława Łojasiewicza Street 11, 30-348 Krakow, Poland; ⊥ Department of Biophysics, Medical University of Lublin, 20-059 Lublin, Poland

## Abstract

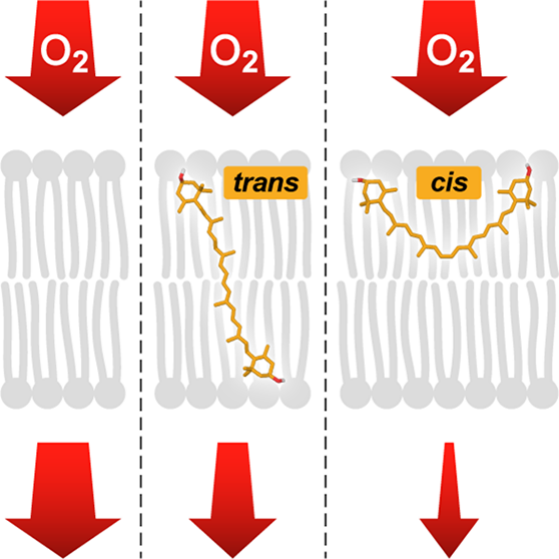

Here, we address the problem of the antioxidant activity of carotenoids in biomembranes. The activity of lutein and zeaxanthin in the quenching of singlet oxygen generated by photosensitization was monitored in lipid vesicles using a singlet oxygen-sensitive fluorescent probe and with the application of fluorescence lifetime imaging microscopy. The antioxidant activity of xanthophylls was interpreted on the basis of electron paramagnetic resonance oximetry results showing that xanthophylls constitute a barrier to the penetration of molecular oxygen into lipid membranes: to a greater extent in the 13-*cis* configuration than in all-*trans*. These results are discussed in relation to the *trans*–*cis* photoisomerization of xanthophylls observed in the human retina. It can be concluded that photoisomerization of xanthophylls is a regulatory mechanism that is important for both the modulation of light filtration through the macula and photoprotection by quenching singlet oxygen and creating a barrier to oxygen permeation to membranes.

Lutein and zeaxanthin belong to a group of polar carotenoid pigments (called xanthophylls) present in both the animal and plant kingdoms and play diverse biological functions, including protection against oxidative damage.^1^ Lutein and zeaxanthin (macular xanthophylls) are present in the human retina, and the possible physiological significance of their heterogeneous distribution is one of the intriguing open questions.^2,3^ The concentration of the macular xanthophylls in the central retina (fovea) is roughly 2 orders of magnitude higher compared to the peripheral regions.^4,5^ Moreover, in the central retina, the concentration of zeaxanthin and *meso*-zeaxanthin is higher than that of lutein, in contrast to the peripheral regions where the lutein fraction is dominant.^5,6^ Several concepts have been proposed to understand the problem of the heterogeneous distribution of macular xanthophylls in the retina, including differences in the photoprotective/antioxidant capacity of lutein and zeaxanthin in the environment of lipid membranes.^2,7^ Recently, we revealed that macular xanthophylls undergo photoisomerization in the human retina, from the molecular configuration all-*trans* to 13-*cis*.^8^ Importantly, this conversion induces the reorientation of xanthophyll molecules in the lipid membranes from vertical to horizontal relative to the plane of the membrane.^8^ In the present work, we address the problem of whether such a different orientation and location of xanthophylls in the lipid bilayer affect their ability to quench singlet oxygen, considered one of the most hazardous molecules in the biosphere, which can initiate oxidative damage to proteins and lipid peroxidation. Singlet oxygen was generated via photosensitization in the surrounding water phase and in the lipid membrane and monitored by a specific, singlet-oxygen-sensitive fluorescence probe present in the lipid vesicles.^9^ The use of fluorescence lifetime imaging microscopy (FLIM) enabled the imaging and analysis of the generation and diffusion of singlet oxygen in a single lipid vesicle. These studies were combined with nanooxymetry based on electron paramagnetic resonance (EPR) spectroscopy, which created the opportunity to gain a unique and precise insight into the antioxidant properties of xanthophylls in the environment of lipid membranes.

Figure 1 shows FLIM images of the same single lipid vesicle illuminated with blue laser light (470 nm) absorbed by the singlet oxygen sensor (SOS) fluorescence probe sensitive to singlet oxygen^10^ or with additional illumination with a 630 nm laser that emits red light absorbed by the toluidine blue (TB) dye, an efficient singlet-oxygen-generating photosensitizer.^9^ Fluorescence emission was analyzed separately in two channels: one selective for SOS and the other selective for TB (see our previous study for a detailed spectroscopic characterization of the system^9^). As seen from the comparison of the fluorescence intensity of the SOS probe, singlet oxygen is efficiently generated in a system containing TB photosensitizer molecules excited with red light to a much greater extent than in the case of excitation with a blue light laser preferentially absorbed by the SOS probe. The fluorescence intensity on the images, recorded in both the TB and SOS channels, shows that singlet oxygen is generated efficiently and concentrated predominantly in the lipid membranes and, to a lesser degree, present also in the water phase inside and outside vesicles (see Figure 1). The ratio of the total fluorescence signals (determined via the integration of emitted fluorescent photons) detected in the SOS channel in a sample excited by the pair of lasers (470 + 630 nm) to the signal recorded with a single laser (470 nm) was proportional to the concentration of singlet oxygen and was used as a quantitative measure of the singlet oxygen level in the system, as generated in the process of TB photosensitization. Figure 2 shows a comparison of the images recorded in the SOS channel with the same lasers but with the liposomes formed with pure 1-palmitoyl-2-oleoyl-*sn*-glycero-3-phosphocholine (POPC) or POPC containing all-*trans* zeaxanthin as an additional lipid membrane component. As seen, the presence of xanthophyll in the membranes significantly reduces the presence of singlet oxygen in the entire system. Figure 3 shows the parameter representing the singlet oxygen level determined for vesicles formed with pure lipid and additionally containing 0.5 mol % carotenoids. As seen, both pigments effectively quench singlet oxygen in the system, and zeaxanthin appears to be slightly more efficient than lutein. Interestingly, 13-*cis* xanthophylls were found to be significantly more effective in antioxidant activity compared to the all-*trans* molecular configuration. To understand such an effect, we used a nanoscale oximetry approach based on the EPR spin label technique.

**Figure 1:**
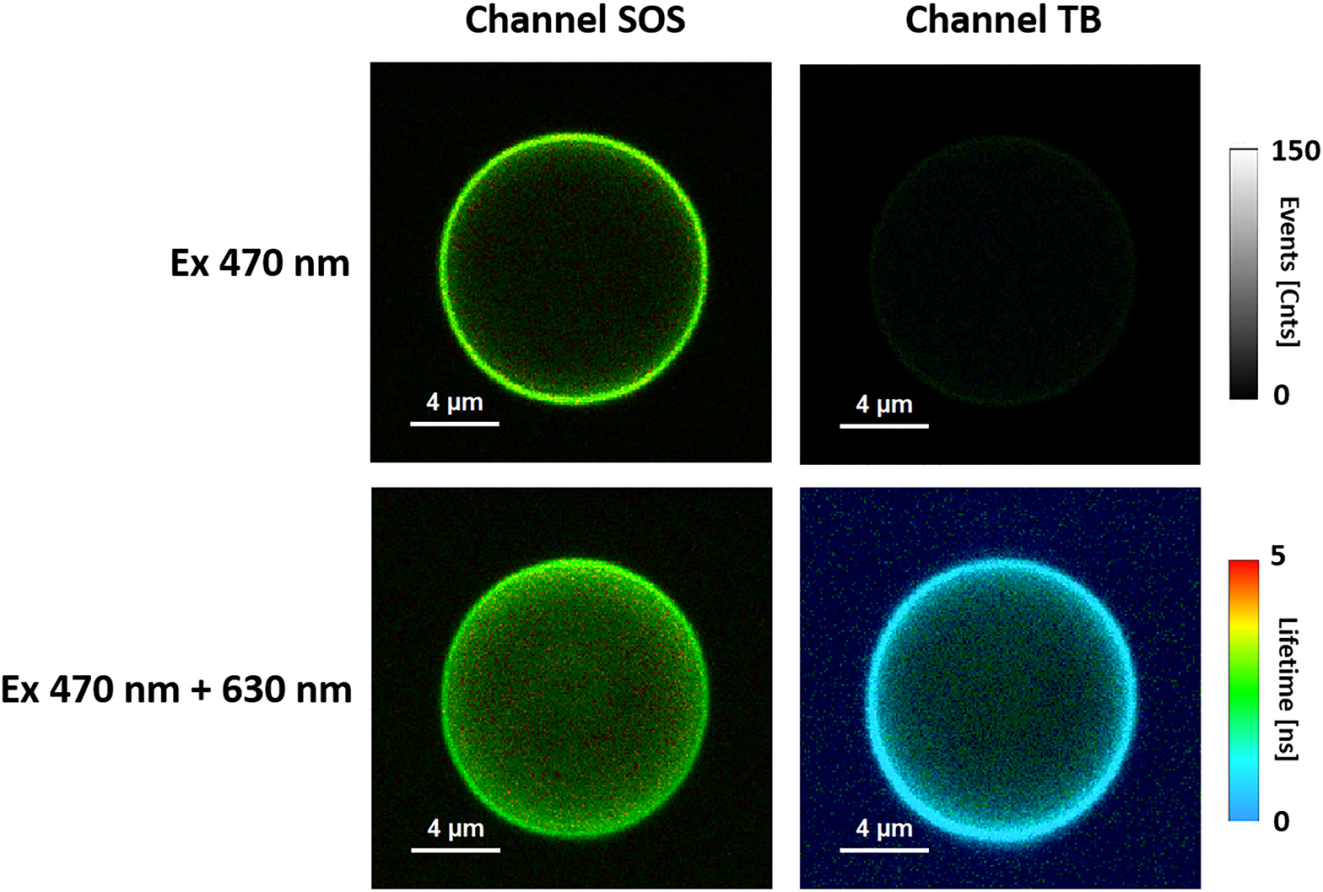
FLIM images of the same liposome equatorial optical cross section. Fluorescence was observed in the channel recording emission from either TB or SOS molecular labels. Excitation was with a 470 nm laser or with two lasers at 470 and 630 nm in pulse interleaved excitation (PIE) mode. Liposomes were formed with POPC.

**Figure 2:**
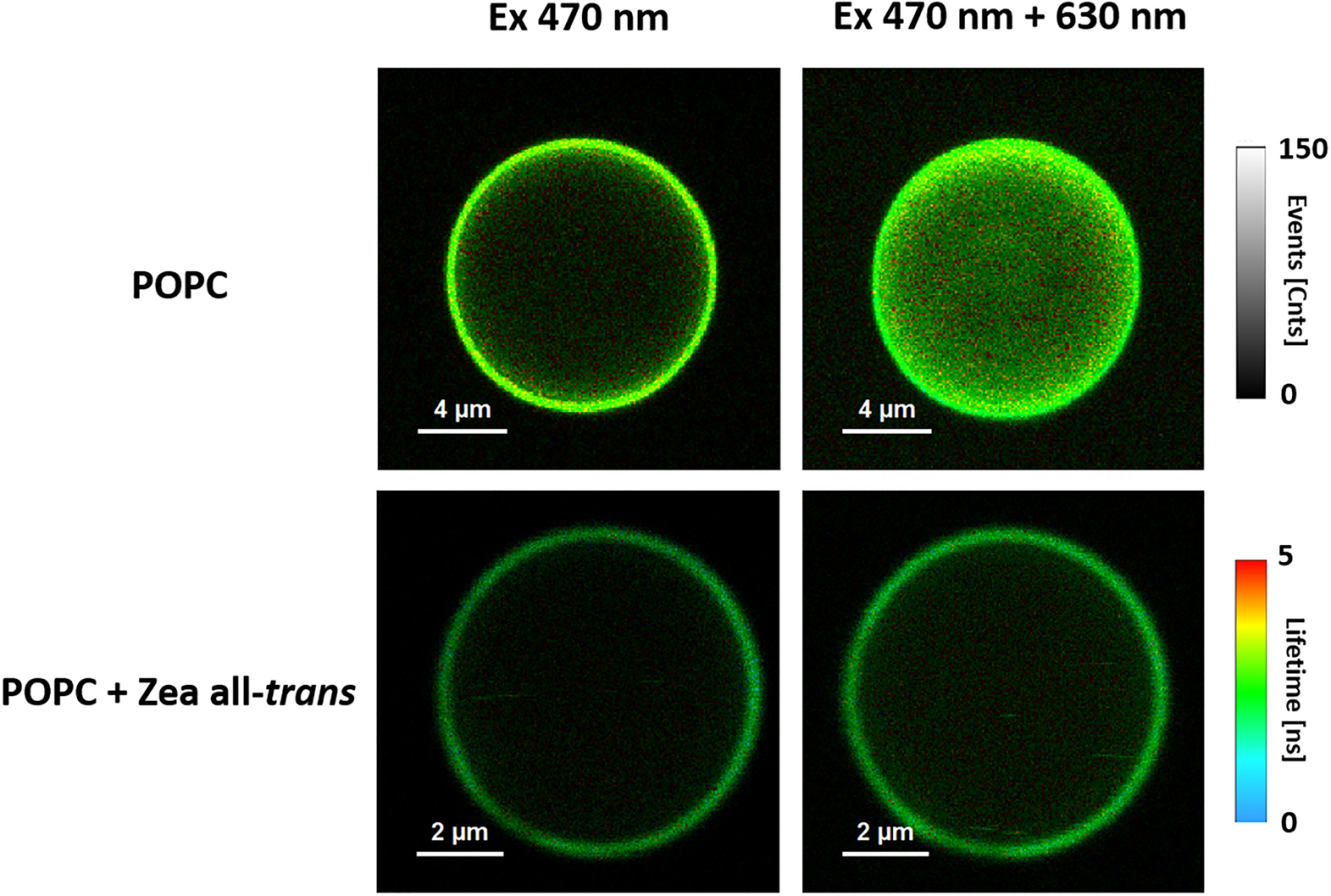
FLIM images of an equatorial optical cross section of the liposome formed with pure POPC or POPC with the addition of 0.5 mol % all-*trans* zeaxanthin. Fluorescence emission was detected in the SOS channel. Excitation was with a 470 nm laser or with two lasers at 470 and 630 nm in PIE mode.

**Figure 3:**
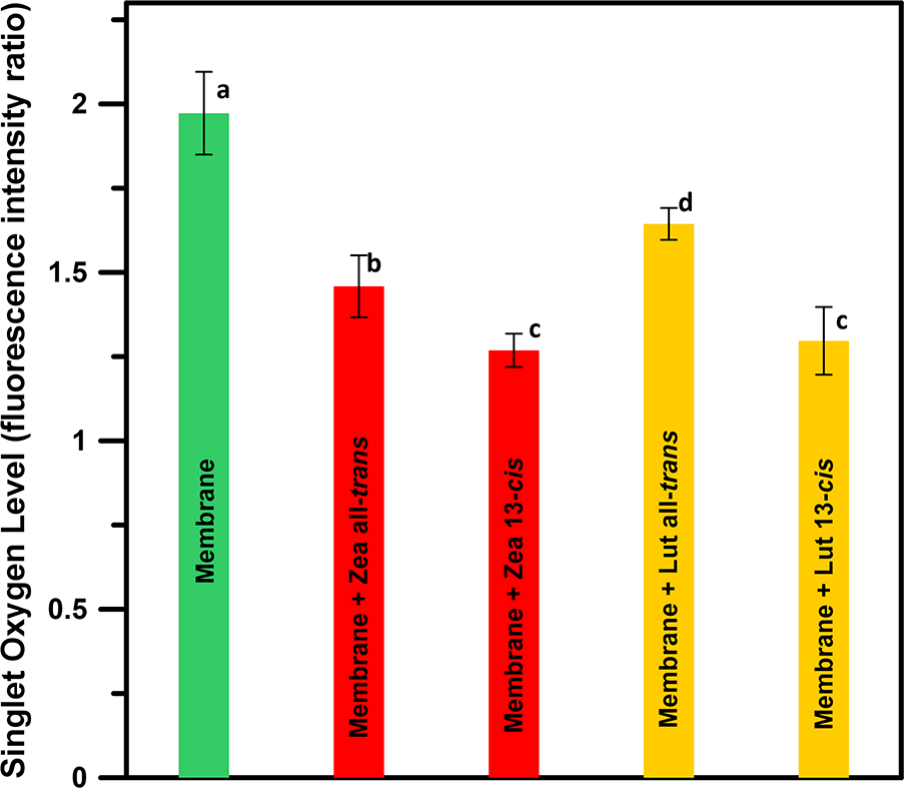
Measure of singlet oxygen levels (proportional to the singlet oxygen concentration) in the system expressed as a total fluorescence signal of SOS (integrated number of emission photons in the SOS channel) in the images of a single liposome recorded with the pair of lasers (470 + 630 nm) divided by the total fluorescence signal of SOS recorded from the same liposome but excited with a single laser (470 nm) inducing fluorescence of the singlet-oxygen-sensitive probe but not absorbed by the TB photosensitizer. The data presented are arithmetic means from five to eight images from two independent preparations ± standard deviation (SD). Values followed by different letters are significantly different from each other (*p* < 0.05): (a) membrane, 1.97 ± 0.12; (b) membrane + all-*trans* zeaxanthin, 1.46 ± 0.09; (c) membrane + 13-*cis* zeaxanthin, 1.27 ± 0.05; (d) membrane + all-*trans* lutein, 1.64 ± 0.05; and (c) membrane + 13-*cis* lutein, 1.30 ± 0.10.

The saturation recovery EPR technique can be applied to monitor the bimolecular collision rate between oxygen and the spin label by oxygen transport parameter (OTP) measurements.^12,13^ It should be noted here that the onset of OTP is not related to active transport across or within the lipid membrane but is a useful monitor of membrane fluidity that reports on the translational diffusion of small molecules, such as oxygen. Figures 4 and 5 present the OTP determined across the lipid membranes formed with POPC and containing additionally incorporated lutein or zeaxanthin in the molecular configuration all-*trans* or 13-*cis*. As seen, both the all-*trans* xanthophylls effectively decrease the OTP within the hydrophobic core of the lipid bilayer, in accordance with the original observation of Subczynski et al. for dimyristoylphosphatidycholine (DMPC) and egg yolk phosphatidycholine (EYPC) membranes containing 10 mol % carotenoids.^14^ Importantly, as revealed in the present study, this effect is pronounced at a relatively low concentration of 1 mol % xanthophyll with respect to lipids (POPC). It is very likely that an overall effect of xanthophylls on the local diffusion-concentration product of the diffusing molecular oxygen is associated with the effect of carotenoids on the structural and dynamic properties of lipid membranes.^15^ Lutein and zeaxanthin are localized in the hydrophobic core of the lipid bilayer,^16^ and their orientation with respect to the membrane plane depends upon a molecular configuration: all-*trans* xanthophylls span the membrane, while the 13-*cis* xanthophylls are oriented in the membrane plane.^8^ On the other hand, the fact that the order parameter determined in the lipid bilayer does not change significantly with such a low xanthophyll concentration (Figure S1 of the Supporting Information) suggests a direct and specific role of lutein and zeaxanthin on the oxidation of OTP in membranes. Although the polyene chain is hydrophobic, it is possible that the incorporation of a xanthophyll molecule into the lipid bilayer modifies the hydrophobic properties of the membrane core and reduces the penetration of oxygen molecules into the membrane. Importantly, the effect of xanthophylls in the 13-*cis* molecular configuration is much greater than in the case of all-*trans*, in the case of both zeaxanthin (Figure 4) and lutein (Figure 5). Such an effect coincides with the singlet oxygen quenching efficiency of xanthophylls, monitored with the fluorescence labeling technique (Figure 3). This suggests that the limited presence of reactive singlet oxygen inside lipid bilayers containing xanthophylls is associated with a higher penetration barrier for molecular oxygen in such modified membranes. On the other hand, the so-called effect of “sacrifice” of xanthophyll molecules, which are oxidized in collisions with singlet oxygen in lipid membranes has to be additionally considered.^9,17^ In the case of a lipid bilayer, oxygen diffuses more freely in the hydrocarbon core of the membrane. The OTP in the central part of the membrane is 2–3 times higher than in the aqueous phase (see OTP profiles for a lipid bilayer formed with DMPC^14^). Thus, a chemical reaction, such as lipid peroxidation, would be expected to occur more readily in the center of the lipid bilayer. In general, our results indicate that both the tested xanthophylls and both of their isomeric forms reduce the OTP in the center of the POPC bilayer (Figures 4 and 5) and reduce the presence of singlet oxygen (Figure 3), which consequently leads to membrane protection against oxidative damage. The fact that the OTP in the central region of the POPC bilayer containing lutein or zeaxanthin is less than that in the pure POPC membrane indicates that either the local oxygen concentration or local oxygen diffusion or both are reduced in the hydrocarbon region of the POPC bilayer in the presence of macular xanthophylls. Additionally, the OTP in the hydrocarbon core is smaller in the case of zeaxanthin compared to lutein. Thus, zeaxanthin could be better suited to play a biological role in protecting biomembranes against oxidative damage. Another important observation is that OTP is particularly reduced in the central region of membranes in the membranes containing xanthophylls in the molecular configuration 13-*cis*. This means that the process of formation of such an isomer in the retina of the human eye exposed to high light^8^ provides additional protection against photo-oxidation. As a result of the hydrophobic mismatch, *cis*-xanthophylls may be oriented horizontally in the POPC membrane in contrast to the all-*trans* configuration.^8^ The average C3–C3′ distance for 13-*cis* zeaxanthin was reported to be ∼21 Å^8^ or ∼24 Å,^18^ whereas the hydrophobic core of the POPC bilayer is ∼30 Å.^19^ Interestingly, a similarly strong effect of 13-*cis* zeaxanthin on OTP, greater than in the case of the all-*trans* isomer, has been reported in the lipid bilayers formed with DMPC, despite the fact that the hydrophobic core of such a lipid bilayer (∼24 Å) is comparable to the distance of polar groups localized on two opposite ends of the xanthophyll molecule in molecular configuration 13-*cis*.^18^ This may suggest that some fraction of the *cis* xanthophyll is oriented in the plane of the DMPC membrane or that the *cis* molecular configuration assures a monomeric state for membrane-bound xanthophylls as a result of a much higher aggregation threshold in the lipid phase compared to *trans* carotenoids.^20^ Despite uncertainty about the exact mechanism underlying this effect, it is important to note that 13-*cis* macular xanthophylls reduce the level of OTP in the hydrophobic core of lipid membranes to a much greater extent than the all-*trans* form. It can therefore be stated that photoisomerization of xanthophylls, observed in the retina under strong light conditions, acts as a regulatory mechanism preparing the entire system for protection against photooxidative damage to photoreceptors and nerve cells. In light of the current and previous findings, it can be concluded that the photoisomerization of macular xanthophylls from the all-*trans* to 13-*cis* molecular configuration serves as a central regulatory mechanism important for not only the blue light filtration but also photoprotection manifested through the antioxidant activity realized by both quenching of singlet oxygen and creating a barrier for the penetration of molecular oxygen into the membranes.

**Figure 4:**
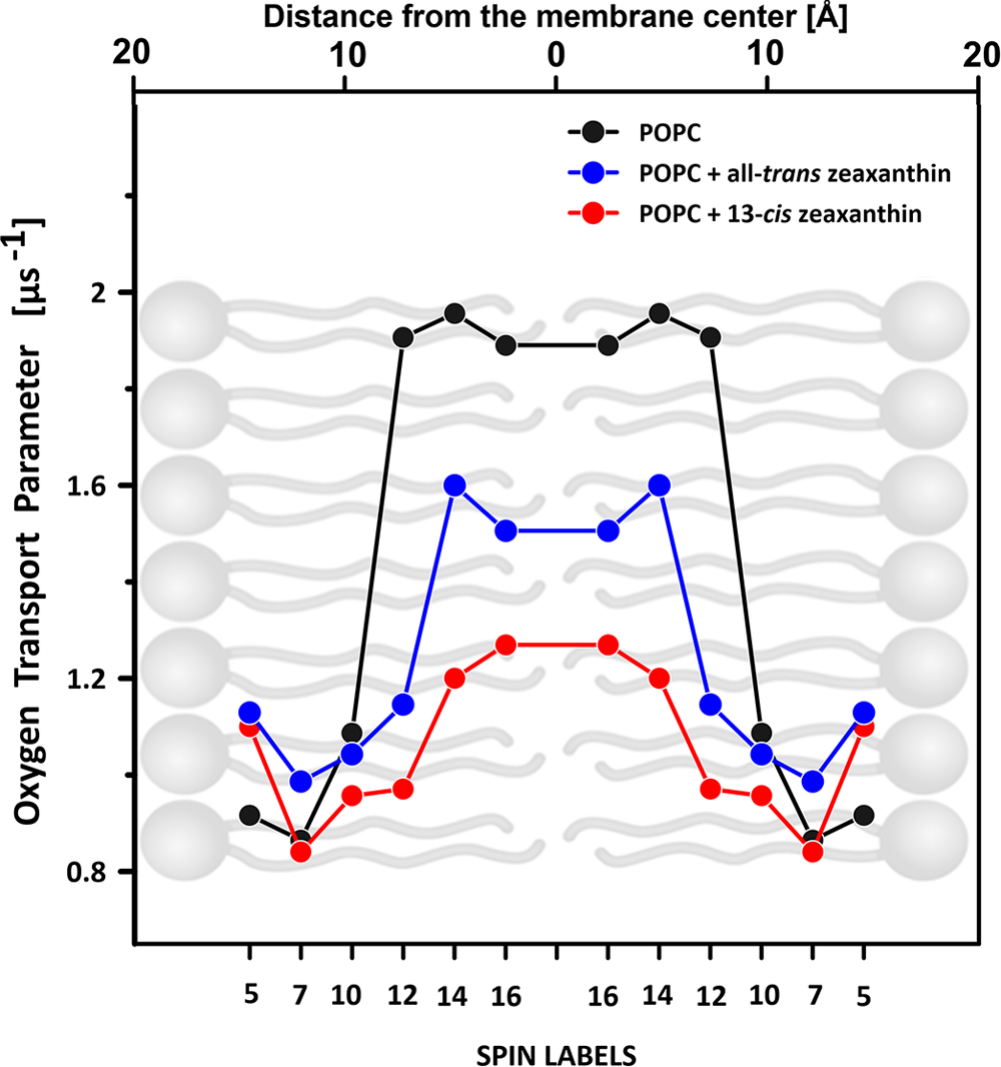
Oxygen transport parameter profile across the lipid bilayer formed with pure POPC or POPC containing 1 mol % zeaxanthin in the molecular configuration all-*trans* or 13-*cis*. The values in the graph represent experiments performed with different *n*-PC spin labels (*n* is shown on the abscissa axis). The relationship is presented superimposed on a lipid bilayer model. Experimental points corresponding to different *n*-PC spin labels represent different experiments performed on separately prepared samples. Differences in the OTP in the central part of the hydrophobic core, determined on the basis of the 14-PC and 16-PC spin labels in each system, express the assay uncertainty. The estimation of the thickness of the POPC membrane, shown above the graph, is based on the molecular dynamics simulation.^11^

**Figure 5:**
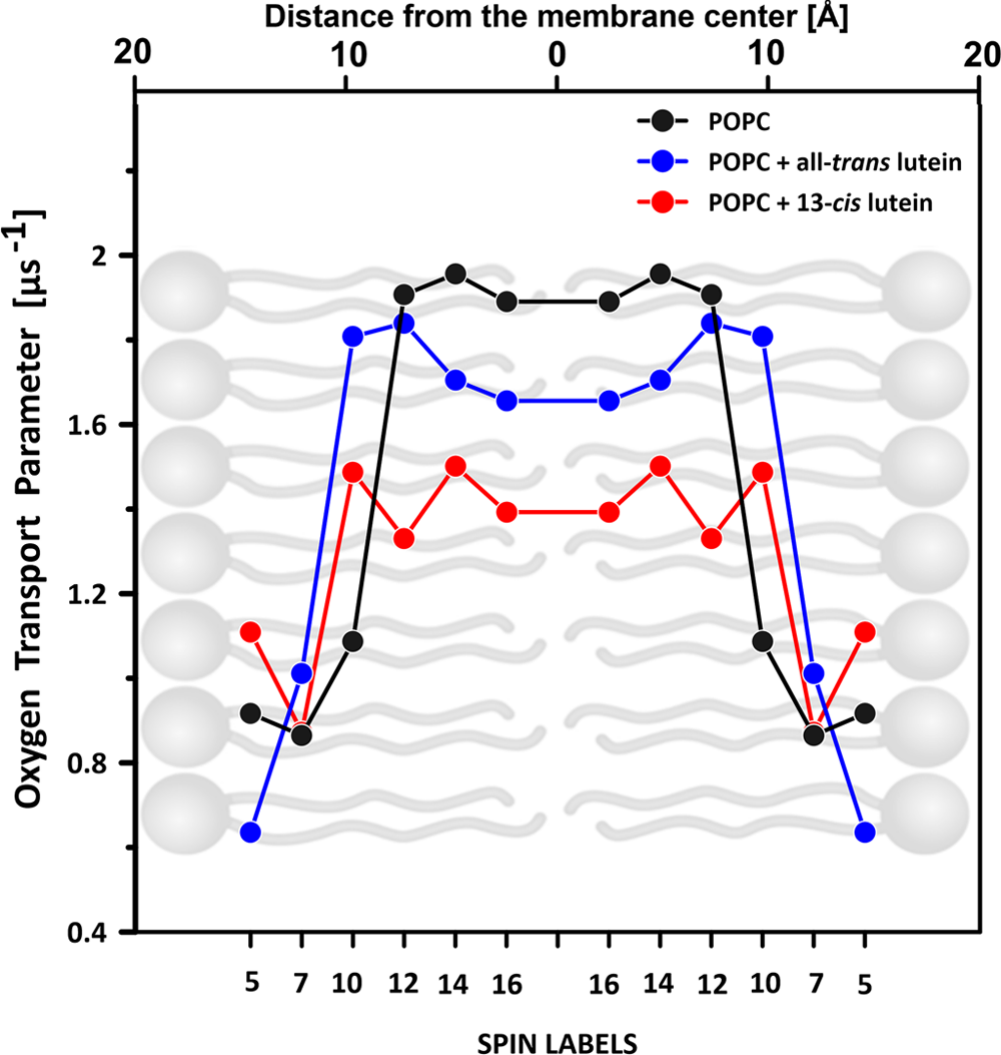
Oxygen transport parameter profile across the lipid bilayer formed with pure POPC or POPC containing 1 mol % lutein in the molecular configuration all-*trans* or 13-*cis*. The values in the graph represent experiments performed with different *n*-PC spin labels (*n* is shown on the abscissa). The relationship is presented superimposed on a lipid bilayer model. Experimental points corresponding to different *n*-PC spin labels represent different experiments performed on separately prepared samples. Differences in the OTP in the central part of the hydrophobic core, determined on the basis of the 14-PC and 16-PC spin labels in each system, express the assay uncertainty. The estimation of the thickness of the POPC membrane, shown above the graph, is based on the molecular dynamics simulation.^11^

## Supporting Information Available

Experimental details and order parameter profile across the hydrophobic core of the POPC membranes formed with pure lipid or containing 1 mol % xanthophyll at molecular configuration all-*trans* or 13-*cis* (Figure S1) (PDF)
