# Supplementary material for: How Do Xanthophylls Protect Lipid Membranes from Oxidative Damage?
Source: J Phys Chem Lett. 2023 Aug 14;14(33):7440–4. doi: 10.1021/acs.jpclett.3c01374 (PMC10461299; doi:10.1021/acs.jpclett.3c01374)
Supplement: Supplementary file 1 — jz3c01374_si_001.pdf [file jz3c01374_si_001.pdf]

Supporting Information

How do Xanthophylls Protect Lipid Membranes from Oxidative Damage?

Renata Welc-Stanowska[a,b], *Rafal Pietras*[c], *Bohun Mielecki*[c,d], *Marcin Sarewicz*[c], *Rafal Luchowski*[a], *Justyna Widomska*[e], *Wojciech Grudzinski*[a], *Artur Osyczka*[c] *and Wieslaw I. Gruszecki*[a,*]

[a] Department of Biophysics, Institute of Physics, Maria Curie-Sklodowska University, 20-031 Lublin, Poland

[b] Institute of Agrophysics, Polish Academy of Sciences, 20-290 Lublin, Poland

[c] Department of Molecular Biophysics, Faculty of Biochemistry, Biophysics and Biotechnology, Jagiellonian University, 30-387 Krakow, Poland

[d] Jagiellonian University, Doctoral School of Exact and Natural Sciences, Prof. St. Łojasiewicza St. 11, 30-348 Krakow, Poland

[e] Department of Biophysics, Medical University of Lublin, 20-059 Lublin, Poland

[*] corresponding author: wieslaw.gruszecki@umcs.pl

Experimental Section

Materials

Crystalline all-*trans* lutein and all-*trans* zeaxanthin were obtained from Extrasynthese. 1-palmitoyl-2-stearoyl-(*n*-doxyl)-*sn*-glycero-3-phosphocholines (*n*-PC spin labels, where n = 5, 7, 10, 12, 14, 16) and 1-palmitoyl-2-oleoyl-sn-glycero-3-phosphocholine (POPC) was purchased from Avanti Polar Lipids Inc. PBS, Singlet Oxygen Sensor Green (SOS) and Toluidine Blue O (TB) were purchased from Sigma Aldrich. Methanol, ethanol, hexane, methyl tert-butyl ether, dichloromethane and sodium thiosulfate were purchased from POCH.

Xanthophyll photoisomerization

All-*trans* zeaxanthin and all-*trans* lutein were dissolved in dichloromethane. Iodine catalyst in hexane was added at a concentration of 2% (w/w) of the xanthophylls. The mixture of iodine and pigment was illuminated for 1 h using a halogen lamp illuminator (150 W) equipped with a band-pass blue filter (B-390, Shimadzu, Japan). In order to remove iodine through a reduction reaction, an aqueous solution of 5% sodium thiosulfate was added. After phase separation, the upper phase was collected and sodium thiosulfate was removed by washing with distilled water. The obtained isomers mixture was dried and dissolved in HPLC mobile phase.

Xanthophyll purification

Isomers of zeaxanthin and lutein were purified chromatographically with the application of Shimadzu LC-20AD system equipped with an SPD-M20A diode array detector and with a C-30 coated, phase-reversed column (YMC GmbH, Germany), internal diameter 4.6 mm, length

250 mm, and particle size 5 μm. A mixture of methanol and methyl tert-butyl ether (95;5, v/v) was used as mobile phase and elution rate was 1 ml/min.

SUV preparation

Small unilamellar vesicles (SUV) for EPR measurements were formed of POPC according to the method described previously. [1] POPC was dissolved in ethanol. Carotenoids as well as n-doxyl labels were added to a lipid solution in ethanol, at a concentration of 1 mol% with respect to POPC. The mixture was dried under gaseous nitrogen and was further kept under vacuum for 1 hour in order to remove traces of an organic solvent. Large multilamellar vesicles were prepared by rehydration with the PBS buffer and vortex shaking for 30 min. In next step, small unilamellar vesicles were prepared by sonication with VCX–130 ultrasonic processor (Sonics Inc., USA) for 15 cycles of 3 s with 100% amplitude with a titanium probe. Sonication process was performed in the water bath, in equilibrium with ice (~0 °C). Final POPC concentration was 0.5 mg/mL.

GUV preparation

Giant Unilamellar Vesicles (GUV) were formed of POPC without or with xanthophylls. The method of formation of GUV was described previously.[2] POPC was dissolved in ethanol and lutein or zeaxanthin in the configuration all-*trans* and 13-*cis* were added to a lipid solution in ethanol, at a concentration of 0.5 mol% with respect to POPC. SOS as a fluorescence probe was added to the buffer at the beginning of GUVs electroformation. The temperature during electroformation was stabilized at 30 °C. Toluidine Blue (TB) as a photosensitizer was added to formed GUVs directly before the measurements. The final SOS and TB concentration in the sample was 5 μM. The method used to prepare GUV allows the formation of liposomes with a

diameter typically in the range of 5-20 μm. Vesicles with a diameter of ~10-15 μm were selected for the measurements.

FLIM measurements

Single GUVs were analyzed with the application of Fluorescence Lifetime Imaging Microscopy (FLIM). FLIM measurements were performed using a two-channel confocal MicroTime 200 (PicoQuant, Germany) system connected to an inverted microscope OLYMPUS IX71. Fluorescence photons were collected with a 100x oil objective (NA 1.3, OLYMPUS UPlanSApo) and a confocal pinhole of 50 μm in diameter was used. The scattered light was removed using a dichroic ZT470/488/640RPC. In order to separate SOS and TB fluorescence signals and observe them with two analyzers, dichroic 620dcxxr (Chroma) was used. Two additional filters: 520/35 (Semrock) and 690/70 (Analysentechnik) were placed in the front of the analyzers set to monitor the fluorescence signal of SOS and TB respectively. Due to the significantly lower quantum efficiency of carotenoid fluorescence emission compared to the SOS fluorescence probe, relatively low concentration of xanthophylls and the methodology of comparing the results, there was no need to correct the emission signal for the endogenous emission of lutein and zeaxanthin. In the first step, GUVs were scanned with 470 nm pulse laser and then both the 470 nm and 630 nm lasers in the Pulsed Interleaved Excitation (PIE) mode of the FLIM system. Singlet oxygen was generated via photo-sensitization of TB. After the interaction of singlet oxygen with SOS, an increase in SOS fluorescence intensity was observed, as tested previously. [3] The ratio of the number of SOS fluorescence photons after and before the excitation of the photosensitizer was calculated as a measure of singlet oxygen level in the sample. All FLIM measurements and imaging were conducted at room temperature 21 ± 1 ºC. The results of measurements were analyzed using SymPhoTime 64 software.

Statistical analysis

One-way analysis of variance (ANOVA) followed by Tukey`s test ($p = 0.05$) was performed with application of Statistica Software (version 13, USA)

EPR measurements

All EPR measurements were made at room temperature (21 ± 1 °C). CW-EPR measurements were carried out in X-band on the Bruker Elexsys E580 spectrometer with SHQ4122 resonator equipped. The resonator was tuned to 9.87 GHz frequency, at which the signal of SL is found between 345 and 357 mT. Other parameters were as follows: microwave power: 2 mW; modulation frequency/amplitude: 100 kHz/0.2 mT; time constant: 10.24 ms; sweep time: 21 s. A spectrum of each sample was averaged repeatedly to the extent where the signal-to-noise ratio was acceptable.

Saturation Recovery (SR) experiments were performed in X-band on a locally built spectrometer with a 1 mm loop-gap resonator [4] with further reconstruction. A pumping microwave pulse of 450 ns length and 100 mW power was applied. Upon saturation, the recovery of the EPR signal was monitored with the use of 14 μW observing microwave power. The signal recovery over time was sampled at 100/200MHz frequency with 4096/2048 time points acquired, depending on the measured relaxation rate. Typically an accumulation of 16-64 million traces was enough for a satisfactory signal-to-noise ratio for each SR curve. Each sample was measured in a gas-permeable TPX capillary which allowed changing from aerobic to anaerobic conditions. [5] The oxygen was removed by blowing the sample inside the cavity with pure nitrogen for at least 20 min before measurement. The SR curves were recorded at a central line of the SL spectrum and were fitted with a single exponential function to obtain spin-lattice relaxation time (T_1). SR EPR method allows observation of the collision rate between

spin labels and oxygen that depends on the local oxygen-diffusion concentration product, named oxygen transport parameter (OTP). [6] The OPT was calculated as difference between spin-lattice relaxation rates (T_1^{-1}) obtained for samples equilibrated with atmospheric air and those equilibrated with nitrogen:

$$\mathrm{OTP(x)} = T_1^{-1}(\mathrm{air, x}) - T_1^{-1}\ (\mathrm{nitrogen, x}) \qquad (1)$$

where, T_1^{-1} is the spin–lattice relaxation rate of the nitroxide moiety of the spin label positioned at the depth x of the membrane equilibrated with air and nitrogen.

The OTP is also proportional to the product of local oxygen diffusion D(x) and local oxygen concentration c(x) at a specific depth in the lipid bilayer:

$$\mathrm{OTP(x)} \sim D\,(\mathrm{x}) \cdot \mathrm{c(\,x)} \qquad (2)$$

where: D(x) is the local oxygen diffusion coefficient and c(x) is the local oxygen concentration at the depth x in the membrane equilibrated with air.

These above two equations (1) and (2) provide the method of obtaining the local oxygen diffusion-concentration product, named by Kusumi et al. as OTP, from the SR EPR measurements. [7] Details of the SR EPR oximetry used in lipid bilayer studies are described in references [5, 8, 9].

The hydrocarbon chain order parameter in the lipid membranes was determined based on the analysis of continuous wave EPR spectra of n-PC spin labels doped into small unilamellar liposomes according to the procedure described in the literature. [10]

REFERENCES

(1) Starzyk, J.; Gruszecki, M.; Tutaj, K.; Luchowski, R.; Szlazak, R.; Wasko, P.; Grudznski, W.; Czub, J.; Gruszecki, W. I. Self-association of amphotericin B: Spontaneous formation of molecular structures responsible for the toxic side effects of the antibiotic. *J. Phys. Chem. B* **2014**, *118*, 13821−13832.

(2) Grudzinski, W.; Nierzwicki, L.; Welc, R.; Reszczynska, E.; Luchowski, R.; Czub, J.; Gruszecki, W. I. Localization and orientation of xanthophylls in a lipid bilayer. *Sci Rep-Uk* **2017**, *7*, Artn 9619.

(3) Widomska, J.; Welc, R.; Gruszecki, W. I. The effect of carotenoids on the concentration of singlet oxygen in lipid membranes. *Biochim Biophys Acta Biomembr* **2019**, *1861* (4), 845-851.

(4) Ilnicki, J.; Koziol, J.; Oles, T.; Kostrzewa, A.; Galinski, W.; Gurbiel, R. J.; Froncisz, W. Saturation recovery EPR spectrometer. *Mol. Phys. Rep.* **1995**, *5*, 203–207.

(5) Subczynski, W. K.; Widomska, J.; Wisniewska, A.; Kusumi, A. Saturation-recovery electron paramagnetic resonance discrimination by oxygen transport (DOT) method for characterizing membrane domains. *Methods Mol Biol* **2007**, *398*, 143-157.

(6) Subczynski, W. K.; Widomska, J.; Raguz, M.; Pasenkiewicz-Gierula, M. Molecular oxygen as a probe molecule in EPR spin-labeling studies of membrane structure and dynamics. *Oxygen (Basel)* **2022**, *2* (3), 295-316. Sarewicz, M.; Pietras, R.; Froncisz, W.; Osyczka, A. Reorientation of cytochrome c(2) upon interaction with oppositely charged macromolecules probed by SR EPR: implications for the role of dipole moment to facilitate collisions in proper configuration for electron transfer. *Metallomics* **2011**, *3* (4), 404-409.

(7) Kusumi, A.; Subczynski, W. K.; Hyde, J. S. Oxygen-transport parameter in membranes as deduced by saturation recovery measurements of spin-lattice relaxation-times of spin labels. *P Natl Acad Sci-Biol* **1982**, *79* (6), 1854-1858.

(8) Subczynski, W. K.; Hyde, J. S. The diffusion-concentration product of oxygen in lipid bilayers using the spin-label T1 method. *Biochim. Biophys. Acta* **1981**, *643* (2), 283-291.

(9) Subczynski, W. K.; Swartz, H. M. EPR oximetry in biological and model samples. . In *Biological magnetic resonance, biomedical EPR – Part A: Free radicals, metals, medicine, and physiology*, Eaton, S. S., Eaton, G. R., Berliner, L. J. Eds.; Kluwer/Plenum, **2005**; pp 229-282.

(10) Subczynski, W. K.; Raguz, M.; Widomska, J. Studying Lipid Organization in Biological Membranes Using Liposomes and EPR Spin Labeling. *Methods Mol. Biol.* **2010**, *606*, 247-269.

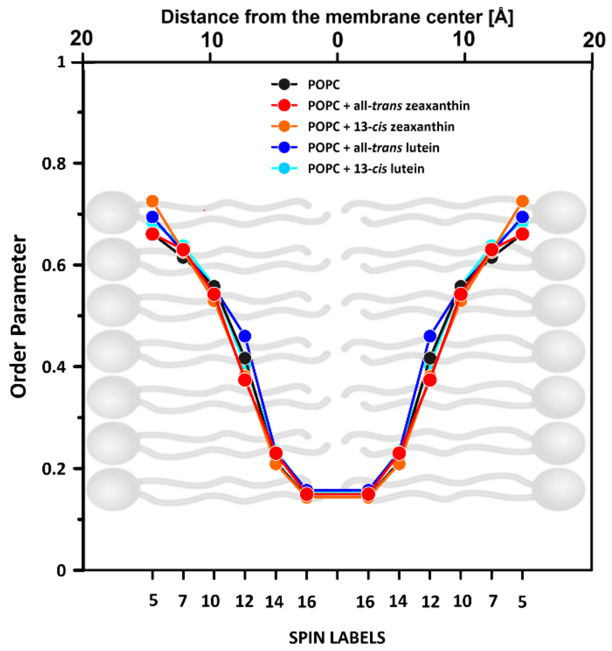

Figure S1 Order parameter profile across the hydrophobic core of the POPC membranes formed with pure lipid or containing 1 mol% xanthophyll at molecular configuration all-*trans* or 13-*cis* (shown in the legend). Order parameters are determined based on continuous wave EPR spectra of n-PC spin labels doped into liposomes (n is shown on the abscissa). Experimental points corresponding to different n-PC spin labels represent different experiments performed on separately prepared samples. Changes between dependencies representing different systems are not significant and fall within the typical uncertainty threshold for determining the ordering parameter based on the spin label technique. The relationship is presented superimposed on a lipid bilayer model.
